# Clinicopathologic and Genomic Features in Triple-Negative Breast Cancer Between Special and No-Special Morphologic Pattern

**DOI:** 10.3389/fonc.2022.830124

**Published:** 2022-03-25

**Authors:** Ying-Zi Li, Bo Chen, Xiao-Yi Lin, Guo-Chun Zhang, Jian-Guo Lai, Cheukfai Li, Jia-Li Lin, Li-Ping Guo, Wei-Kai Xiao, Hsiaopei Mok, Chong-Yang Ren, Ling-Zhu Wen, Fang-Rong Cao, Xin Lin, Xiao-Fang Qi, Yang Liu, Ning Liao

**Affiliations:** ^1^ Department of Breast, Guangdong Provincial People’s Hospital, Guangdong Academy of Medical Sciences, Guangzhou, China; ^2^ Medical College, Shantou University, Shantou, China; ^3^ The Second School of Clinical Medicine, Southern Medical University, Guangzhou, China; ^4^ OrigiMed Co. Ltd., Shanghai, China

**Keywords:** Chinese breast cancer, triple negative breast cancer, special type, mutation landscape, PD-L1 (22C3)

## Abstract

**Background:**

Triple-negative breast cancer (TNBC) is refractory and heterogeneous, comprising various entities with divergent phenotype, biology, and clinical presentation. As an aggressive subtype, Chinese TNBC patients with special morphologic patterns (STs) were restricted to its incidence of 10-15% in total TNBC population.

**Methods:**

We recruited 89 patients with TNBC at Guangdong Provincial People’s Hospital (GDPH) from October 2014 to May 2021, comprising 72 cases of invasive ductal carcinoma of no-special type (NSTs) and 17 cases of STs. The clinical data of these patients was collected and statistically analyzed. Formalin-fixed, paraffin-embedded (FFPE) tumor tissues and matched blood samples were collected for targeted next-generation sequencing (NGS) with cancer-related, 520- or 33-gene assay. Immunohistochemical analysis of FFPE tissue sections was performed using anti-programmed cell death-ligand 1(PD-L1) and anti-androgen receptor antibodies.

**Results:**

Cases with NSTs presented with higher histologic grade and Ki-67 index rate than ST patients (NSTs to STs: grade I/II/III 1.4%, 16.7%,81.9% vs 0%, 29.4%, 58.8%; p<0.05; Ki-67 ≥30%: 83.3% vs. 58.8%, p<0.05), while androgen receptor (AR) and PD-L1 positive (combined positive score≥10) rates were lower than of STs cases (AR: 11.1% vs. 47.1%; PD-L1: 9.6% vs. 33.3%, p<0.05). The most commonly altered genes were *TP53* (88.7%), *PIK3CA* (26.8%), *MYC* (18.3%) in NSTs, and *TP53* (68.8%), *PIK3CA* (50%), *JAK3* (18.8%), *KMT2C* (18.8%) in STs respectively. Compared with NSTs, *PIK3CA* and *TP53* mutation frequency showed difference in STs (47.1% vs 19.4%, p=0.039; 64.7% vs 87.5%, p=0.035).

**Conclusions:**

In TNBC patients with STs, decrease in histologic grade and ki-67 index, as well as increase in PD-L1 and AR expression were observed when compared to those with NSTs, suggesting that TNBC patients with STs may better benefit from immune checkpoint inhibitors and/or AR inhibitors. Additionally, lower TP53 and higher PIK3CA mutation rates were also found in STs patients, providing genetic evidence for deciphering at least partly potential mechanism of action.

## Introduction

Triple-negative breast cancer (TNBC) is a special molecular subtype of breast cancer (BC) with unique biological and clinicopathologic characteristics, defined as estrogen receptor (ER)-, progesterone receptor (PR)-, and human epidermal growth factor receptor 2 (HER-2) - negative, representing 15-20% of BC cases (1, 2). Compared to other BC subtypes, TNBC is associated with a high histologic grade and strong invasiveness, lacking the opportunity for endocrine and targeted therapy due to the lack of corresponding targets (3). Chemotherapy is currently the main treatment, but its curative effect is unsatisfactory (4). At the histologic and genetic levels, TNBC represents a group of highly heterogeneous BCs, with the highest BRCA mutation rate among BC subtypes, particularly BRCA1 (5, 6). Moreover, invasive ductal carcinoma of no-special type (NSTs) also includes some special histologic subtypes with a special morphologic pattern (STs), such as medullary carcinoma, metaplastic carcinoma, and apocrine carcinoma (7–12). Although their immunophenotypes are triple-negative, their morphology, prognosis, and response to treatment are quite different (13). In recent years, TNBC has received extensive attention from a clinical and pathologic aspect. Targeted drugs for molecular typing and multiple signaling pathways have been extensively studied. In 2011, Lehmann et al. indicated the molecular classification of TNBC by DNA microarray firstly (14). However, for STs of TNBC, there are few studies on the combination of histologic examination results with genetic information, limited to its 10-15% incidence in TNBC.

At present, molecular-level research on BC has made great progress and gradually entered the era of precision treatment. This study aimed to identify the biological characteristics of the main TNBC histologic subtypes with genomic differences constituting important prognostic factors. The genomics of NSTs were compared to that of STs, and the panel, covering 520 cancer-related genes, was analyzed using capture-based ultra-deep targeted sequencing technology. A total of 89 TNBC cases were identified. The results of the genetic changes in the patients were combined with detailed histologic examination and PD-L1, AR test results for analysis.

## Materials and Methods

### Patient Selection

In this study, 89 hormone receptor (HR)-/HER2- patients with various tumor stages (stages I-IV), diagnosed at Guangdong Provincial People’s Hospital (GDPH) between October 2014 and May 2021, were enrolled. ER and PR expression in BC specimens were routinely evaluated at the Department of Pathology in GDPH, following the 2010 and 2013 American Society of Clinical Oncology (ASCO)/College of American Pathologists (CAP) guidelines (15, 16). The HR status was determined as negative when immunohistochemistry (IHC) results of ER and PR were both <1%, while the HER2 status was determined as negative when IHC staining results were negative (0 to 1+) or equivocal (2+), and no amplification was identified by fluorescence *in situ* hybridization. This study was approved by the Ethics Committee of GDPH, and all patients provided written informed consent for translational research. Sequencing assays, blinded to the clinicopathologic parameters of the Clinical Laboratory Improvement Amendments-certified Burning Rock Biotech (Guangzhou, China), were performed.

### Histologic and Clinicopathologic Criteria

In total, 89 patients’ clinicopathologic data were collected, comprising histologic subtypes, morphological features, age at diagnosis, tumor characteristics, Ki-67 index, tumor infiltrating lymphocytes (TILs), AR, and PD-L1 expression. Histological features of all available TNBC samples were centrally reviewed by the pathologists in GDPH.

**Figure 1:**
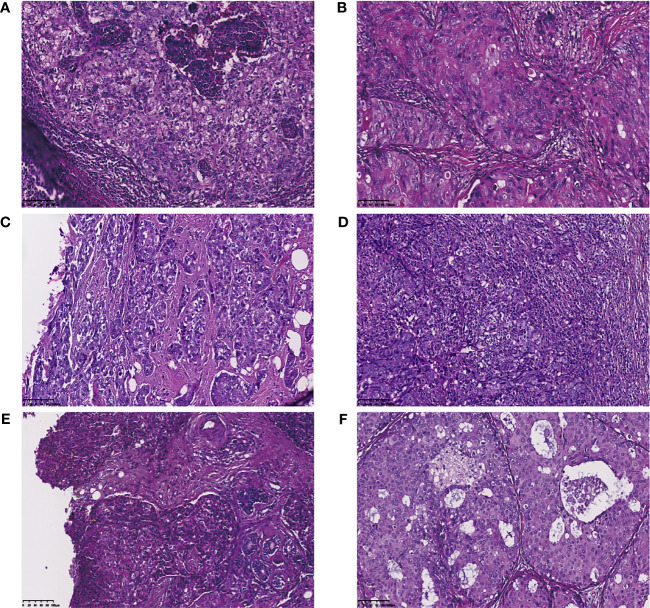
Invasive ductal carcinoma of no-special type and special morphologic pattern of triple negative breast cancers. **(A)** Invasive ductal carcinoma; **(B)** Apocrine carcinoma (Carcinoma with apocrine differentiation); **(C)** Metaplastic breast carcinoma; **(D)** Medullary carcinoma; **(E)** Glycogen-rich clear cell carcinoma; **(F)** Mixed type.

According to the 2012 and 2019 World Health Organization classification, BCs were classified as invasive ductal carcinoma NSTs and STs (special morphologic patterns, including metaplastic carcinoma, apocrine carcinoma, glycogen-rich clear cell carcinoma, and medullary carcinoma) (**Figure 1**) (17–19), of which the predominant histologic subtype is the pattern with the highest percentage. Metaplastic carcinoma is defined as a carcinoma with squamous differentiation or spindle cell morphology (20). Apocrine carcinoma is defined as nuclear enlargement with prominent nucleoli and abundant, granular, eosinophilic cytoplasm (21). Glycogen-rich clear cell carcinoma is characterized by the presence of neoplastic cells with a glycogen-abundant clear cytoplasm (periodic acid-Schiff-positive, diastase-sensitive) (19). Medullary carcinoma always has the indistinct borders of the “pushing” type, giving the tumor a syncytial or sheet-like appearance. The tumor cells of this subtype are large and pleomorphic, with large nuclei, prominent nucleoli, and numerous mitoses. The prominent lymphoplasmacytic infiltrate at the periphery of the tumor is an important feature of medullary carcinoma (22).

### Immunohistochemistry

Immunohistochemical staining of formalin fixed, paraffin embedded (FFPE) tumor tissue sections were performed using anti-PD-L1 antibodies (clone 22C3, Cat#M3653, DAKO, 1:50 dilution; OrigiMed, Shanghai) and anti-AR antibodies (clone AR441, Mouse monoclonal, DAKO, 1:50 dilution, Gene Tech, Shanghai). All slides were counterstained with hematoxylin. PD-L1 expression was interpreted as a combined positive score (CPS), which was defined as the number of PD-L1-positive cells (including tumor cells, lymphocytes, and macrophages) divided by the total number of tumor cells, and then multiplied by 100. According to the 2021 National Comprehensive Cancer Network (NCCN) guidelines (23), the threshold for PD-L1 positivity was set at ≥10%. A positive AR status was defined as average of ≥1% positive tumor nuclei, which is the same cut-off follows the 2010 ASCO/CAP guidelines for ER and PR, frequently chosen by other studies.

### Tumor-Infiltrating Lymphocytes Evaluation

Tumor infiltrating lymphocytes (TILs) were evaluated on hematoxylin and eosin (HE)-stained tissue sections at a magnification of ×20–40 with a ×10 ocular, according to the recommendations of an International Tumor Infiltrating Lymphocytes Working Group (24, 25). “High”TILs were defined as a tumor with 40-90% stromal TILs, presented as percentage of the stromal areas alone, and “Intermediate” TILs were defined as 10-40%, while “Low” TILs were defined as 0-10%.

### Comprehensive Genomic Profiling

Next-generation sequencing library preparation and sequence data analyses were conducted according to a previous study (26, 27). Genomic profiling was performed using a panel of 520 cancer-related genes (OncoScreen Plus; Burning Rock Biotech, Guangzhou, China). Whole exons of 312 genes and critical exons, introns, and promoter regions of the remaining 208 genes were captured. Ten mL of peripheral blood was collected. Events were then classified as germline or somatic, depending on their presence in the matched normal set (white blood cells) of events.

### Sequence Data Analysis

Sequence data were mapped to the human genome (hg19) using BWA aligner v.0.7.10. Local alignment optimization, variant calling, and annotation were performed using GATK v.3.2 and VarScan v.2.4.3. Variants were filtered using the VarScanfpfilter pipeline, with loci with depths less than 100 filtered out. At least five supporting reads were needed for insertions or deletions, while eight supporting reads were required for single-nucleotide variations (SNVs). According to the ExAC, 1000 Genomes, dbSNP, and ESP6500SI-V2 databases, variants with population frequencies over 0.1% were grouped as single-nucleotide polymorphisms (SNPs) and excluded from further analysis. The remaining variants were annotated using ANNOVAR and SnpEff v.3.6. DNA translocation analysis was performed using the Factera v.1.4.3.

### Statistical Analysis

All data were summarized by frequency and percentage for categorical variables including mutation detection rate and distribution of mutation types. SPSS software version 26.0 (IBM Corp., Armonk, NY, USA) and R 3.5.1 (R Core Team, Vienna, Austria) were used for statistical analyses. Pearson’s chi-squared (χ2) test and Fisher’s exact test were used to test the pairwise correlation among clinicopathologic features, mutation detection rate, and distribution of mutation types. Statistical significance was set at p < 0.05.

## Results

### Clinicopathologic Features of the Patients

In total, tumor tissues of 89 patients with invasive TNBC were examined, which were divided into the NSTs (N=72) and STs (N=17) groups according to their histologic type (**Figure 1**). The clinicopathologic features of the patients are summarized in **Table 1**. All 89 patients were female, with a median age at diagnosis of 49 (range 22-81) years. The premenopausal status rate was 58.4% (of 52/89). The majority of patients included in this cohort presented with an early TNM stage. As shown in **Table 1**, the NSTs group had a higher histologic grade (NST to ST: grade I, 1.4%; grade II, 16.7%; and grade III, 81.9% vs. grade I, 0%; grade II, 29.4%; and grade III, 58.8%; p<0.05) than the ST group according to the 8th edition of the American Joint Committee on Cancer (AJCC) staging system (18). No significant association was found between age at onset, menopausal status, lymph node status, tumor size, and TNM stage between the two groups.

**Table 1:** Clinicopathologic characteristics of the GDPH TNBC cohort (N=89).

Variables	TNBC		No-special Type		Special Type		p
	N=89	No. (%)	N=72	No. (%)	N=17	No. (%)	
Age							0.813
[median, range]	49	[22-81]	49	[23-75]	46	[22-81]	
≤50 years	52	58.4	43	59.7	9	52.9	
>50 years	37	41.6	29	40.3	8	47.1	
Menopausal status							1
Pre-menopause	49	55.1	40	55.6	9	52.9	
Post-menopause	40	44.9	32	44.4	8	47.1	
T-stage							0.428
T1	39	43.8	33	45.8	6	35.3	
T2	40	44.9	30	41.7	10	58.8	
T3	6	6.7	6	8.3	0	0.0	
T4	4	4.5	3	4.2	1	5.9	
N-stage							0.604
N0	48	53.9	39	54.2	9	52.9	
N1	22	24.7	16	22.2	6	35.3	
N2	13	14.6	11	15.3	2	11.8	
N3	6	6.7	6	8.3	0	0.0	
M-stage							1
M0	88	98.9	71	98.6	17	100.0	
M1	1	1.1	1	1.4	0	1.0	
Pathologic stage							0.621
I	27	30.3	22	30.6	5	29.4	
II	42	47.2	32	44.4	10	58.8	
III	19	21.3	17	23.6	2	11.8	
IV	1	1.1	1	1.4	0	0.0	
Histologic grade							0.022^#^
I	1	1.1	1	1.4	0	0.0	
II	17	19.1	12	16.7	5	29.4	
III	69	77.5	59	81.9	10	58.8	
NA	2	2.2	0	0.0	2	11.8	
Ki-67(%)							0.040^#^
[median, range]	65	[5-95]	70	[10-95]	50	[5-90]	
<14%	7	7.9	3	4.2	4	23.5	
≥14%	81	91.0	68	94.4	13	76.5	
<30%	18	20.2	11	15.3	7	41.2	0.048^#^
≥30%	70	78.7	60	83.3	10	58.8	
NA	1	1.1	1	1.4	0	0.0	
AR							0.002^#^
Positive (≥1%)	16	18.0	8	11.1	8	47.1	
Negative (<1%)	59	66.3	50	69.4	9	52.9	
NA	14	15.7	14	19.4	0	0.0	
TILs							0.092
Low (0-10%)	36	40.4	27	37.5	9	52.9	
Intermediate (10-40%)	14	15.7	14	21.5	0	0.0	
High (40-90%)	32	36.0	24	33.3	8	47.1	
NA	7	7.9	7	9.7	0	0	
Histologic type							<0.001^#^
IDC	72	81.0	72	100.0	0	0.0	
Mixed	6	6.7	0	0.0	6	35.3	
AC	5	5.6	0	0.0	5	29.4	
MC	4	4.5	0	0.0	4	23.5	
MBC	1	1.1	0	0.0	1	5.9	
GRCC	1	1.1	0	0.0	1	5.9	

The p-value was calculated using the Pearson χ2 test and Fisher’s exact test; #, p-value<0.05. TNBC, triple-negative breast cancer; TILs, tumor-infiltrating lymphocyte; AR, androgen receptor; IDC, infiltrating ductal carcinoma; Mixed, mixed histologic; AC, apocrine Carcinoma; MC, medullary carcinoma; MBC, metaplastic breast carcinoma.

Ki-67 index data were available from 88 patients, with a median Ki-67 index of approximately 65% (ranging from 5% to 95%). In the NST group, the number of patients with a high Ki-67 index (≥30%) was significantly higher than that in the ST group (83.3% vs. 58.8%, p<0.05). Meanwhile, androgen receptor (AR) expression data were available from 75 patients, showing that the number of AR-positive NSTs patients was lower than that in the STs group (11.1% vs. 47.1%, p<0.01).

Furthermore, the PD-L1 IHC results of 67 qualified tumor samples were analyzed, including 52 NST and 15 ST cases (**Table 2**). The percentage of PD-L1 positivity was 14.9% (10/67) in 67 patients with TNBC. Compared the expression levels of PD-L1 between the two groups, a PD-L1 score cut-off of 10 for positive was selected and found that the proportion of STs with PD-L1 CPS greater than or equal to 10 was higher than that of NSTs (33.3%; 9.6%, p=0.038). Considering PD-L1 as an immune biomarker, these results may suggest that ST TNBC patients might benefit from immunotherapy over NST TNBC patients. The stromal TILs for each case were also evaluated, and no significant differences were found between the two groups.

**Table 2:** Characteristics of PD-L1 expression in the GDPH TNBC cohort (N=67).

Variables	TNBC		No-special		Special		p
	N=67	No. (%)	N=52	No. (%)	N=15	No. (%)	
PD-L1(cut-off=10)							0.038^#^
Negative (<10)	57	85.1	47	90.4	10	66.7	
Positive (≥10)	10	14.9	5	9.6	5	33.3	

p-value was calculated using the Pearson χ2 test and Fisher’s exact test; #, p-value<0.05.

### Genomic Alteration Landscape of TNBC

**Figure 2:**
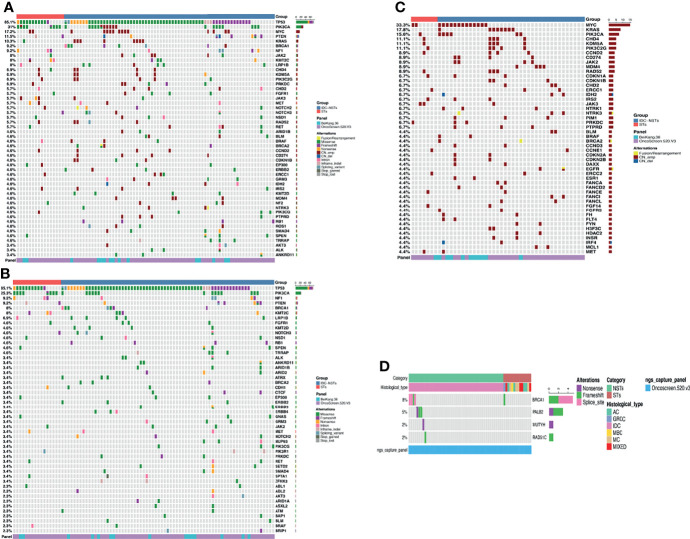
The landscape of genetic alterations in TNBC. Top 50 genomic alterations are shown in the Oncoprint. Different colors denote different types of alterations and different clinicopathologic features. **(A)** Summary of the features of the genomic alteration of the 89 patients with TNBC. Tumor samples were grouped according to histologic types as: no-special type (NSTs, n = 72) and special type (STs, n = 17). The top bar shows the histologic type of each patient; the side bar (rows) summarizes the percentage of tumors with alterations in each gene (left) and alteration composition for each gene in the entire cohort (right). **(B)** Summary of the features of the genomic mutation of the 89 patients with TNBC. Tumor samples were grouped according to histologic types as: no-special type (NSTs, n = 72) and special type (STs, n = 17). The top bar shows the histologic type of each patient; the side bar (rows) summarizes the percentage of tumors with mutations in each gene (left), and the mutation composition for each gene in the entire cohort (right). **(C)** Summary of copy number variations and Fusion in the 45 patients with TNBC who carry copy number variations. Tumor samples were grouped according to histologic types as: no-special type (NSTs, n = 38) and special type (STs, n = 7). The top bar shows each patient’s histologic type; the side bar (rows) summarizes the percentage of tumors with variation in each gene (left) and alteration composition for each gene (right), in the entire cohort. **(D)** Summary of germline mutation of the 62 patients with TNBC. Tumor samples were grouped according to histologic types as: no-special type (NSTs, n = 48) and special type (STs, n = 14). The side bar (rows) summarizes the percentage of tumors with mutation in each gene (left) and alteration composition for each gene (right), in the entire cohort. TNBC, triple-negative breast cancer; NST, no-special type; ST, special type; indel, insertions or deletions; LGR, large genomic rearrangement; CN_amp, copy number amplification; CN_del, copy number deletion.

A total of 794 alterations, including 513 single-nucleotide variants (SNVs), 252 copy number (CN) amplifications, five copy number (CN) deletions, 14 insertions or deletions (Indels), nine fusions, and one large genomic rearrangement were detected in 285 genes from 89 patients. As shown in **Figure 2**, *TP53*(85.1%) and *PIK3CA* (31%) were the top-ranked altered genes in this cohort, in which missense mutations were the predominant alterations. Further, alterations detected in the cohort included *MYC* (17.2%), *PTEN* (11.5%), and *KRAS* (10.3%). Besides, the vast majority of highest-frequency copy number alterations were seen in 43 patients (48.3%), including amplifications in *MYC* (33.3%), *KRAS* (17.8%), *PIK3CA* (15.6%), and *CHD4* (11.1%). Based on the available data from 62 patients (paired white blood cells and tumor samples were sequenced using a panel consisting of 520 cancer-related genes, including 62 cancer susceptibility genes to interrogate germline and somatic alterations, respectively), the analysis revealed that 10 patients carried pathogenic germline mutations (10/62, 16.1%) for *BRCA1* (5/62, 8.0%), *PALB2* (3/62, 4.8%), *MUTYH* (1/62, 1.6%) and *RAD51C* (1/62, 1.6%). Except for one ST patient, all other patients carrying pathogenic germline mutations were of the NSTs subtype.

### Differentially Mutated Genes Between TNBC NST and ST Patients

**Figure 3:**
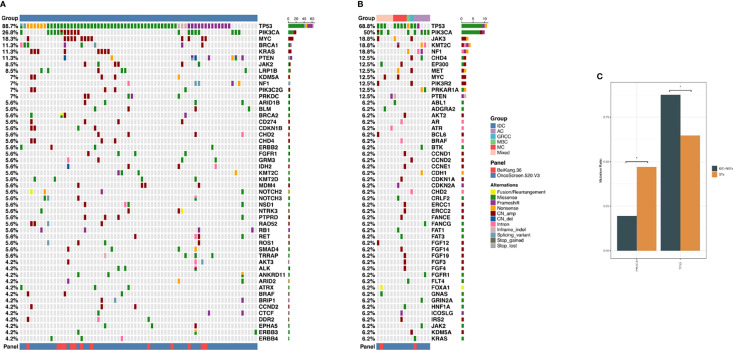
**(A)** Summary of genomic features of the 72 NST cases of TNBC patients. **(B)** Summary of genomic features of the 17 ST cases of TNBC patients. **(C)** Differentially mutated genes between NSTs and STs TNBC patients. The X-axis represents the specific genes. The Y-axis represents the percentage of samples with mutations in a specific gene. * indicated p values <0.05. TNBC, triple-negative breast cancer; NST, no-special type; ST, special type.

The differentially altered genes in TNBC NST and ST tumors were compared. As shown in **Figure 3**, the top five altered genes were *TP53* (88.7%), *PIK3CA* (26.8%), *MYC* (18.3%), *BRCA1* (11.3%), and *KRAS* (11.3%) in NSTs, and *TP53* (68.8%), *PIK3CA* (50%), *JAK3* (18.8%), *KMT2C* (18.8%) and *NF1* (18.8%) in STs. Considering mutations of oncogenic genes, *TP53* (87.5%), *PIK3CA* (19.4%), *PTEN* (9.7%), and *BRCA1* (8.3%) were most frequently mutated in NSTs, while *TP53* (64.7%), *PIK3CA* (47.1%), and *NF1* (11.8%) were the most frequently mutated in STs. *TP53* and *PIK3CA* were found to be differentially mutated between NSTs and STs. The mutation frequency of *TP53* was higher in TNBC NSTs than in STs, while *PIK3CA* mutations were more frequent in STs (87.5% vs. 64.7%, p=0.035; 19.4% vs. 47.1%, p=0.039, respectively). Moreover, *BRCA1* somatic mutations were only identified in six (8.3%) NST TNBC patients. Copy number variant analysis of 38 NST patients and 7 STs patients revealed that *MYC* (34.2%), *KRAS* (21.1%), and *PIK3CA* (15.8%) were more frequently amplified in NSTs. In STs, however, the top-ranked copy number amplification genes were *JAK3* (11.8%), *MYC* (11.8%), and *PIK3R2* (11.8%).

**Figure 4:**
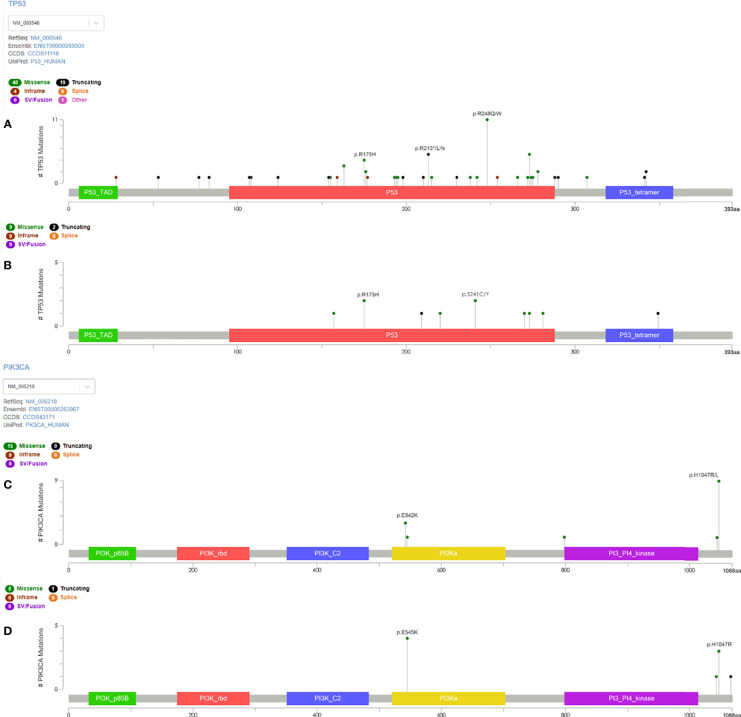
Lollipop diagram of the *TP53* and *PIK3CA* domains with the mutation location identified in TNBC patients. Different types of mutations were colored by different colored dots, and each colored dot represents one mutation. The length of the lollipop represents the number of patients harboring a specific variant. **(A)** The type and location of *TP53* mutation in NST patients. **(B)** The type and location of *TP53* mutation in ST patients. **(C)** The type and location of *PIK3CA* mutation in NST patients. **(D)** The type and location of *PIK3CA* mutation in ST patients. TNBC, triple-negative breast cancer; NST, no-special type; ST, special type.

The alteration types and mutation sites of *TP53* and *PIK3CA* in the TNBC-NST and ST groups were compared, studied, and are illustrated in **Figures 3**, **4**. Sixty-three NST TNBC patients (63/72, 87.5%) and 11 STs patients (11/17, 64.7%) had *TP53* mutations, which occurred in several exons (exons 4-10). Missense mutations were the dominant mutation form in both groups (39/63, 61.9%; 9/11, 81.8%), followed by frameshift and nonsense mutations (13/63, 20.6%; 6/63, 9.5%; 1/11, 9.1%; 1/11, 9.1%, respectively). The mutation sites of *TP53* were also different between the two groups. Among NST TNBC patients, p.R248Q/W (11/63, 17.5%), p.R213*/fs/L (5/63, 7.9%), and p.R175H (4/63, 6.3%) accounted for the highest proportion of all mutation sites, whereas p.R175H and p.S241C/Y (2/11, 18.2%) mutation sites occupied the highest proportion, without p.R248 site-related mutations in the ST group.

Regarding *PIK3CA*, a total of 24 mutations occurred in 22 patients, including 14 NST and eight ST patients; and one patient had double mutations in each group. *PIK3CA* mutations occurred in exons 10, 16, and 21 in the NSTs, and in exons 10 and 21 in ST patients. In both groups, missense mutations were the primary alteration type, followed by copy number amplification. In the NST group, p.H1047R (8/14, 57.1%) mutation sites accounted for the highest proportion, followed by p.E542K (3/14, 21.4%) substitutions. In the ST group, p. E542K (4/8, 50%) and p.H1047R (3/8, 37.5%) mutation sites accounted for the highest proportion.

### Mutation Pathway Analysis in NST and ST Patients

**Figure 5:**
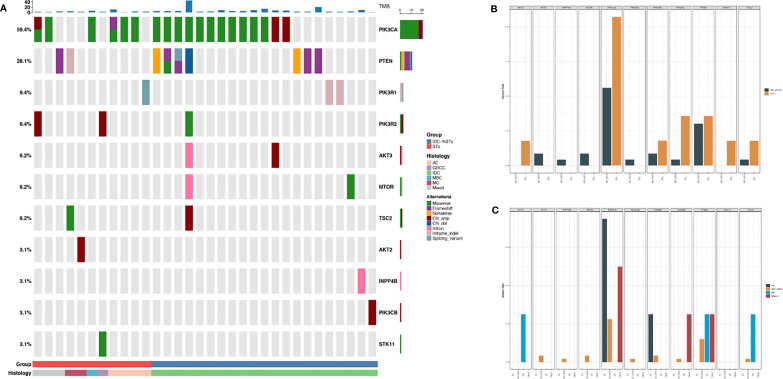
Kyoto Encyclopedia of Genes and Genomes analysis reveals distinct pathways in NSTs and STs tumors of TNBC. In total, 36.2% of the NSTs patients and 78.6% of the STs patients had at least one clinically relevant genomic alteration in genes involved in the PI3K-AKT signaling pathways, respectively. **(A)** Summary of the features of the PI3K-AKT signaling pathways genomic alteration of the 32 patients with TNBC. Tumor samples were grouped according to histologic types as: no-special type (IDC-NSTs, n=21) and special type (STs, n=11). **(B)** Comparison of the features of the PI3K-AKT signaling pathways genomic alteration between NSTs and STs TNBC patients. **(C)** Comparison of the features of the PI3K-AKT signaling pathways genomic alteration among STs TNBC patients. TNBC, triple-negative breast cancer; NST, no-special type; ST, special type; TMB, tumor mutation burden.

For further analysis, the molecular signatures database was used to analyze the distinct pathways that were affected by NSTs and STs. It revealed the major signaling pathways affected by genomic alterations in the ST patients as PI3K (**Figure 5**, 36.2% vs. 78.6%, p= 0.006). Although the presence of other signaling pathways in the two groups was diverse (cell cycle, HIPPO, TGF-beta, etc.), the statistical differences were insignificant. An in-depth pathway analysis within ST patients was performed, illustrating that patients with mixed histologic type presented lower PI3K pathway mutation levels than medullary carcinoma and apocrine carcinoma, but were still higher than NSTs. Mutations in genes involved in the PI3K pathway, detected in our cohort, included *AKT2* (n=1), *AKT3* (n=2), *INPP4B* (n=1), *MTOR* (n=2), *PIK3CA* (n=19), *PIK3CB* (n=1), *PIK3R1* (n=3), *PIK3R2* (n=3), *PTEN* (n=9), *STK11* (n=1), and *TSC2* (n=2). Alterations in *AKT3* (n=2), *INPP4B* (n=1), *MTOR* (n=2), *PIK3CA* (n=6), *PIK3CB* (n=1), *PIK3R1* (n=2), *PIK3R2* (n=1), *PTEN* (n=7), and *TSC2* (n=1) were detected in 58 patients with NST tumors. Five patients with NSTs had concurrent alterations in at least two genes. Meanwhile, *AKT2* (n=1), *PIK3CA* (n=10), *PIK3R1* (n=1), *PIK3R2* (n=2), *PTEN* (n=2), *STK11*(n=1), and *TSC2* (n=1) alterations were detected in 14 patients with ST tumors. Among these, three patients had at least two gene alterations.

Collectively, these results indicate that NST and ST tumors are molecularly distinct based on the difference in the number and distribution of somatic alteration types, as well as the signaling pathways affected by these alterations.

## Discussion

TNBC is clinically and molecularly heterogeneous and highly refractory and belongs to a mixed sub-category. It is a collective term for the remaining BCs after excluding ER, PR, and HER2-positive patients, accounting for 15%-20% of all BCs. In addition to the majority of invasive ductal carcinomas of NSTs, TNBC also includes a small number of STs, which have special morphological characteristics. According to the Cancer Genome Atlas (TCGA) and the Molecular Taxonomy of Breast Cancer International Consortium (METABRIC) cohorts, STs TNBC patients had poorer prognosis than NSTs (**Supplementary Figure 1**). The Surveillance, Epidemiology, and End Results (SEER) database was utilized to determine the differences between TNBC-NSTs and STs, regarding survival, overall survival, and disease-specific survival rates (28). However, adjuvant therapy for TNBC NSTs and STs is indifferently represented by chemotherapy, without effective prognostic markers or therapeutic targets. To develop precision therapy, the molecular classification of TNBC is defined by DNA microarrays, gene sequence expression, mRNA expression, and IHC (29–31). This research explored the differences in clinicopathologic and molecular characteristics between the NSTs and the STs of Chinese patients with TNBC, looking for relevant markers that may be helpful in the development of precise and individualized systemic drugs for TNBC.

The 89 patients with TNBC were divided into two groups according to pathologic type for comparison. TNBC-NSTs and STs were similar in terms of the age of onset, tumor size, and TNM stage. Interestingly, the histologic grade and Ki-67 index of the NSTs were significantly higher than those of the STs. The histologic grades of invasive BCs are based on the differentiation of tumor cells, which is an important factor in predicting the prognosis of patients with BC and tumor aggressiveness. As confirmed by the NCCN guidelines, a higher histologic grade is an unfavorable factor for BC, which may require more aggressive adjuvant treatments. Ki-67 is a proliferation biomarker that is considered an independent predictive and prognostic factor for the management of BC (32, 33). BCs with high Ki-67 expression have proved to respond better to chemotherapy, and are associated with poor prognosis. Higher histologic grade and Ki-67 index of the NSTs may indicate that the prognosis of NSTs is worse than that of STs.

Immunotherapy with anti-PD-1/PD-L1 agents has emerged as a new treatment modality for TNBC in recent years (34, 35), introducing a novel therapeutic field. PD-L1 expression has been recognized as the most important biomarker for predicting the response to immunotherapy in cancers (36). Mittendorf et al. described that approximately 20%-30% of breast tumors express PD-L1, especially TNBC (37). In this study, the percentage of PD-L1 positivity was 14.9% in 67 TNBC patients. Compared with NSTs, the positive percentage of PD-L1 in STs was higher. These results suggest that ST patients may benefit more from treatment targeting the PD-L1 pathway. Besides, targeted therapy of AR has become another major hotspot in TNBC treatment. Research showed that STs patients presented significantly higher AR-positive rates than NSTs, which indicates that these patients are more likely to benefit more from AR inhibitors (38, 39). Multiple clinical studies have examined immunotherapy for TNBC. For advanced TNBC, based on the results of the Impassion130 and KEYNOTE-355 trials, Atezolizumab and Pembrolizumab have been approved in combination with chemotherapy as first-line treatments for PD-L1 positive advanced TNBC (40–42). For early TNBC, the results of KEYNOTE-522 trial set a new standard for early TNBC immunotherapy, and the combination chemotherapy of Pembrolizumab has been approved by the FDA for neoadjuvant therapy in patients with high-risk TNBC (43). Updates to the GeparNUEVO trial also confirmed the positive significance of neoadjuvant immunotherapy. To date, neither AR inhibitors (bicalutamide and enzalutamide) nor androgen biosynthetic inhibitor (abiraterone) exhibited significant anticancer activity against patients with AR positive metastatic TNBC (clinical benefit rates range from 19% to 35%) (44). Several clinical studies are under way.

For developing precision therapy, molecular information has some instructive significance. While the genomic landscape of TNBC is heterogeneous and complex, it was shown that Chinese patients with TNBC display a similar mutation spectrum to that reported in studies conducted in other countries, and *TP53* (85.1%), *PIK3CA* (31%), and *MYC* (17.2%) are some of the most frequent alterations. Further studies performed to compare gene mutation profiling of NSTs to those of STs have found that the NSTs had higher frequencies of *TP53* mutation than STs, while the *PIK3CA* mutation frequency was higher in the STs than in the NSTs. The tumor suppressor gene, *TP53*, is the most frequently mutated gene in human cancer (45, 46). Patients with BC with a somatic *TP53* mutation have poor prognosis, which is consistent with the present research. Unfortunately, *TP53* mutations are not presently targetable (47), and their predictive capacity for various therapies for BC has not been thoroughly examined. *TP53* mutations are frequently found in *BRCA1*-associated human BCs (48–50). Except for one ST patient, six patients carrying *BRCA1* mutations were found in NSTs in this study. Describing the mutation site of *TP53* in the two groups separately, p.R248Q/W, p.R213*/fs/L, and p.R175H had the highest proportion in the NST group, while p.R175H and p.S241C/Y mutation sites had higher frequencies in the ST group. Although there are differences in the mutational profiling of *TP53* between the two groups, the relevance of the mutation hotspots identified in the GDPH patients to the treatments for BC remains to be determined. Recently, Bert Vogelstein et al. successfully identified a bispecific single chain diabody (scDb) highly specific to the common TP53 mutations, and confirmed its mechanism of activating T cells to exert anti-tumor effects (51). Nevertheless, gene therapies, targeted tumor vaccines, and anti-cancer agents for *TP53* alteration are still in the early stages of clinical trials, including APR-246, which is a targeted drug for three hot-spot mutations of the *TP53* gene (R273, R175, and R248) (52, 53). Their effectiveness has not been proven in clinical trials. Therefore, authorities such as the United States Food and Drug Administration (FDA) have not approved any treatment for *TP53* alteration yet. For TNBC patients who have BRCA defection or mutation, PARP inhibitors can block BRCA1/2-mediated homologous-recombinant based DNA double-strand break repair and promote tumor cell apoptosis. In the OlympiAD study, Olaparib, the PARP inhibitor had an improvement in median OS and 3-year survival rate compared with traditional chemotherapy for metastatic TNBC (54). This study led to olaparib’s approval from the FDA and EMA for the treatment of HER2-negative metastatic breast cancer patients with BRCA 1/2 mutations, opening the door to TNBC-targeted therapy. In 2021, Interim data from the OlympiA study were released, which suggesting that one-year adjuvant therapy with Olaparib alone can obviously reduce the risk of recurrence and death in HER2-negative early-stage breast cancer patients with gBRCA mutation (55). Olaparib has been accepted as the standard adjuvant therapy for high-risk HER2-negative early-stage breast cancer patients with gBRCA mutations.

From a molecular perspective, mutations in *PIK3CA* genes are found significantly more frequently in STs than in NSTs. Alteration types were dominated by missense mutations in both groups. Nevertheless, copy number amplification accounted for a larger proportion of NSTs than that in STs, indicating that the genome of the NSTs was unstable. Consistent with previous studies, the majority of *PIK3CA* mutations occurred in three hotspot sites, namely E542K and E545K, in the helical domain and H1047R, in the kinase domain (56, 57). In the NST group, p.H1047R (57.1%) mutation sites accounted for the highest proportion, while p.E542K (50%) mutation sites were the most frequently altered in the ST group. For HR-positive metastatic BC, the present study showed that the p.H1047R mutation might be a potential biomarker of sensitivity to everolimus, an mTOR inhibitor (58). Notably, the FDA-approved *PI3K* inhibitor, alpelisib, is highly recommended in *PIK3CA*-mutated HR-positive BC according to the most recently updated guidelines (23). Although the TNBC ST patients were negative for ERs and PRs, 50% of the ST patients still harbored *PIK3CA* mutations; thus, it is rational to assume the potential effect of alpelisib in the treatment strategy for the STs. Considering AKT as a downstream effector protein for PI3K, phase II trial LOTUS and PAKT tested highly selective oral pan-AKT inhibitors Ipatasertib and Capivasertib in combination with paclitaxel in the treatment of metastatic TNBC, with significant improvement in PFS (59, 60). In the FAIRLANE Phase II trial, adding Ipatasertib with neoadjuvant chemotherapy would significantly increase the pCR rate compared with adding placebo (61).

This study has several limitations. First, the relatively small number of cases in the two morphologic groups renders the analysis performed exploratory and hypothesis-generating. Secondly, the presence of somatic genetic alterations affecting 520 genes was surveyed. Hence, the possibility of additional differences between the NSTs and STs of TNBC being present if whole exome or whole genome sequencing was performed cannot be excluded. Incorporation of comprehensive genomic profiling into TNBC may shed light on potential therapeutic opportunities for both targeted drugs and immune checkpoint inhibitors. Additionally, prognostic data for patients with TNBC in the GDPH queue was not provided in this study. Although the higher Ki-67 index, histologic grades, and proportion of *TP53* mutations suggested that the NST patients had poor prognoses, there was no reliable evidence to prove the relationship between the NSTs and STs. The STs have multiple pathological types, considering that some TNBC subtypes may exhibit different somatic cytogenetic changes. Resultantly, it is necessary to further study the genomic pattern of specific TNBC subgroups.

In conclusion, this study has shown significant differences in clinicopathologic features and gene signatures between Chinese NST and ST TNBC patients. The increased PD-L1 and AR expression, as well as elevated *PIK3CA* mutation frequency in the STs group implied that potential treatments might be available for these patients. Further studies with lager sample size and clinical outcomes data will be needed to explore the possibility to use immune checkpoint inhibitors, AR inhibitors and PIK3CA mutation-related treatments in TNBC patients with ST.

## Data Availability Statement

The datasets presented in this study can be found in online repositories. The names of the repository/repositories and accession number(s) can be found below: NODE (http://www.biosino.org/node) with accession OEP002982 (http://www.biosino.org/node/project/detail/OEP002982).

## Ethics Statement

The studies involving human participants were reviewed and approved by GDREC2014122H. The patients/participants provided their written informed consent to participate in this study.

## Author Contributions

NL, BC, and Y-ZL conceived, designed and supervised the study. Y-ZL, X-YL, J-LL, C-YR, L-ZW, F-RC, XL, L-PG, X-FQ, and YL collected the data, analyzed and interpreted them. YL, BC, X-YL, NL, G-CZ, J-GL, W-KX, CL, and HM wrote and revised the manuscript. All authors read and approved the final manuscript for submission.

## Funding

This study received support from the Fundamental Research Funds for the Central Universities (y2syD2192230, BC); the National Natural Science Foundation of China (81902828, BC); High-level Hospital Construction Project of Guangdong Provincial People’s Hospital (DFJH201921, BC); Guangdong Medical Research Foundation (B2019039, BC); Natural Science Foundation of Guangdong Province (2016A030313768, NL; 2018A030313292), and Research Funds from Guangzhou Municipal Science and Technology Project (201707010418, NL).

## Conflict of Interest

Authors X-FQ and YL are employed by OrigiMed Co. Ltd.

The remaining authors declare that the research was conducted in the absence of any commercial or financial relationships that could be construed as a potential conflict of interest.

## Publisher’s Note

All claims expressed in this article are solely those of the authors and do not necessarily represent those of their affiliated organizations, or those of the publisher, the editors and the reviewers. Any product that may be evaluated in this article, or claim that may be made by its manufacturer, is not guaranteed or endorsed by the publisher.

## Supplementary Material

Supplementary Figure 1 Kaplan-Meier survival curves based on no-special type infiltrating ductal carcinoma and special types of triple negative breast cancer patients. **(A)** Disease free status of patients in the Cancer Genome Atlas cohort. **(B)** All patients in the Cancer Genome Atlas cohort. **(C)** All patients in the Molecular Taxonomy of Breast Cancer International Consortium cohort.

Supplementary Figure 2 Immunohistochemical staining of programmed death-ligand 1(PD-L1) in triple- negative breast cancer (Magnification 200×). **(A)** Negative control of PD-L1 expression by immunohistochemistry. **(B)** Low PD-L1 expression in triple- negative breast cancer tumor samples. **(C)** High PD-L1 expression in triple- negative breast cancer tumor samples.

Supplementary Figure 3 Hematoxylin and eosin staining of tumor-infiltrating lymphocytes (TILs) in triple-negative breast cancer (Magnification 200×). **(A)** Low-grade TILs. **(B)** Intermediate-grade TILs. **(C)** High-grade TILs.
