# Supplementary material for: Clinicopathologic and Genomic Features in Triple-Negative Breast Cancer Between Special and No-Special Morphologic Pattern
Source: Front Oncol. 2022 Mar 25;12:830124. doi: 10.3389/fonc.2022.830124 (PMC8989735; doi:10.3389/fonc.2022.830124)
Supplement: Supplementary Figure 1 — Kaplan-Meier survival curves based on no-special type infiltrating ductal carcinoma and special types of triple negative breast cancer patients. (A) Disease free status of patients in the Cancer Genome Atlas cohort. (B) All patients in the Cancer Genome Atlas cohort. (C) All patients in the Molecular Taxonomy of Breast Cancer International Consortium cohort. [file DataSheet_1.docx]

Supplementary Material

## Supplementary Figures


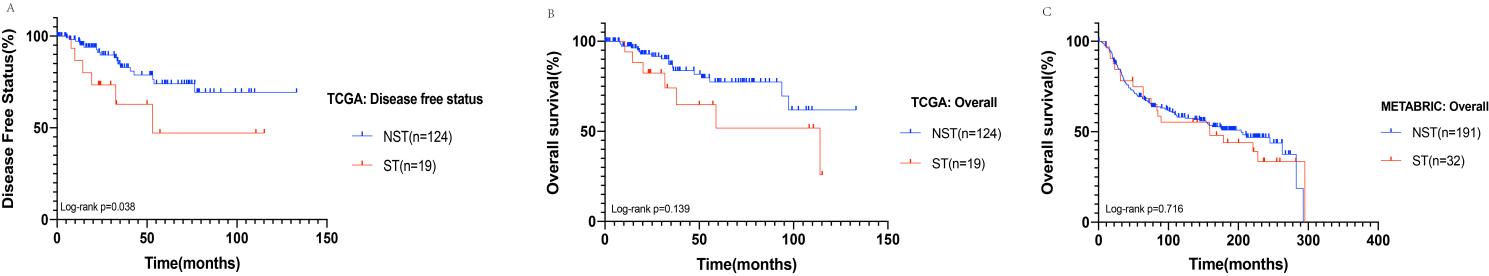


**Supplementary Figure 1.** Kaplan-Meier survival curves based on no-special type (IDC) and special types of triple negative breast cancer patients

A. Disease free status of patients in the TCGA cohort.

B. All patients in the TCGA cohort.

C. All patients in the METABRIC cohort.


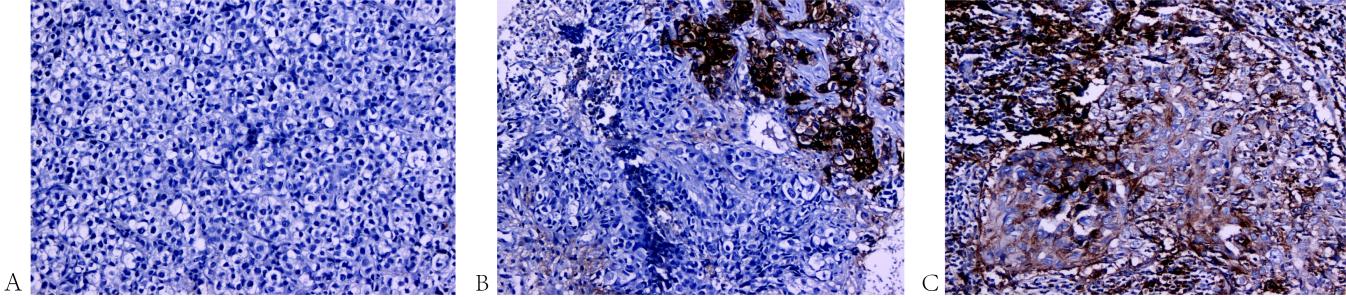


**Supplementary Figure 2.** Immunohistochemical staining of PD-L1 in triple negative breast cancer (Magnification 200×).

A. Negative control of PD-L1 expression by IHC.

B. Low PD-L1 expression in TNBC tumor samples.

C. High PD-L1 expression in TNBC tumor samples.


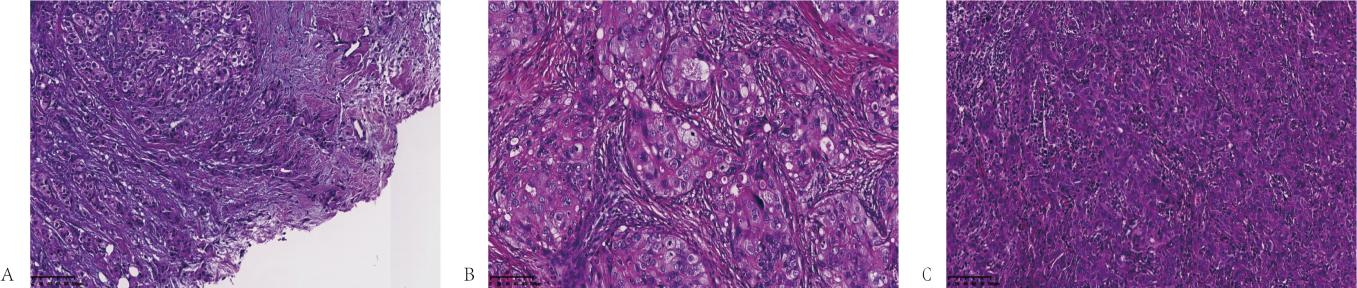


**Supplementary Figure 3.** Hematoxylin and eosin staining of TILs in triple negative breast cancer (Magnification 200×).

A. Low-grade TILs.

B. Intermediate-grade TILs.

C. High-grade TILs.
